# Assessing Physiological and Psychological Factors Contributing to Stress among Medical Students: Implications for Health

**DOI:** 10.3390/ijerph192416822

**Published:** 2022-12-15

**Authors:** Fawzia Al-Rouq, Alanoud Al-Otaibi, Alanoud AlSaikhan, Maha Al-Essa, Sarah Al-Mazidi

**Affiliations:** 1 Physiology Department, Faculty of Medicine, King Saud University, Riyadh 11362, Saudi Arabia; 2 Family Medicine Department, King Khalid University Hospital, Riyadh 12372, Saudi Arabia; 3 Radiology Department, King Khalid University Hospital, Riyadh 12372, Saudi Arabia; 4 Physiology Department, Faculty of Medicine, Imam Mohammad Ibn Saud Islamic University, P.O. Box 5701, Riyadh 11432, Saudi Arabia

**Keywords:** physiology, stress, medical students, pandemic

## Abstract

**Background:** Physiological responses to stress disturb internal homeostasis, leading to serious health consequences. Medical students experience high stress levels that should be managed promptly to prevent stress-related impacts on students’ health and education. **Aim:** This study aims to identify the relationship between stress factors, general health, and academic performance. **Methods:** This study recruited 421 medical students of all academic years. Participants completed an online survey assessing stress levels using a validated 10-item Perceived Stress Scale. Stress was also compared with students’ health and academic performance. **Results:** We found that 93.6% of our sample experienced moderate to severe stress, and 31% reported increased stress due to the coronavirus disease (COVID-19) pandemic. Except for internship students, stress significantly decreases as students progress each academic year (*p* < 0.05). Students with higher GPAs and with comorbidities are more stressed. Comorbidities were primarily reported in students in their final years of education with a 4% lower GPA than healthy students. Although we had three stress-related themes (general, academic, and pandemic), students’ perceptions of stress factors were primarily academically related. **Conclusions:** Students experience high stress levels in their final educational years, which might increase the risk of health issues and low academic performance. It is essential to innovate stress-coping strategies specially designed for medical students and mandatorily provided by all medical colleges and to educate students on the effects of stress on their health.

## 1. Introduction

Stress is a complex psychological and physiological experience that leads to serious health consequences. The framework of stress has four elements, including stimulus, stressor (stress factors), stress response (immediate physiological response), and stress effects (prolonged stress consequences), that vary according to the severity and type of stress stimulus [1]. Physiologically, experiencing stress at a certain level is beneficial for anticipating future challenges and establishing adaptation. However, excessive stress, also known as distress, activates the defense response of the central nervous system, which disturbs the hypothalamic-pituitary-adrenal (HPA) axis. The HPA axis controls neuroendocrine hormones, and prolonged disturbance to the HPA axis might lead to cardiovascular, endocrine, and metabolic disorders [2,3]. Moreover, the HPA axis affects sleep centers, which might lead to sleep disorders affecting a student’s cognitive function, mood, and academic performance [4,5,6]. Cognition and memory are also affected by altered hippocampus dysfunction due to chronic stress [7].

Medical students experience more stress than their peers in non-medical fields [8,9]. The high rates of stress in medical students are due to the high academic load, increased competition, and long working and studying hours. Medical students also experience more sleep deprivation than other students, especially in the clinical years, affecting their cognitive skills, academic performance, and, eventually, patient care [10,11].

Stress affects students’ coping behaviors, such as eating habits, smoking, and substance abuse [9,12]. This might reflect the high prevalence of obesity reported in health colleges, which was associated with lower academic performance and higher stress levels [13,14]. Stress was also linked to blood type and comorbidities such as insulin resistance and hypertension [15].

Pandemics are a recent factor contributing to stress in medical students. Studies reported that the coronavirus disease (COVID-19) pandemic has significantly increased stress among medical students [16]. A survey conducted during COVID-19, including samples from different colleges, showed that medical students were the most stressed, especially females [17,18].

Previous studies reported that stress differs between medical students according to their academic year. Some stated that first-year students are more stressed, whereas others reported that stress increases as students progress through their academic years [6,18]. Stress and sleep deprivation increase as students progress academically and are associated with lower academic performance [19].

Along with the physiological effects of stress on cognition and memory, stress affects students’ academic performance, which is measured by grade point average (GPA). Students with higher stress had lower GPAs, lower self-coping strategies, and less psychosocial support. However, other studies reported that GPA was not significantly correlated with stress among students and did not differ within academic years.

Stress and its physiological effects have attracted attention in this field, with one factor leading to the other in a cause-and-effect vicious cycle. There are different scales to measure stress levels among medical students. Some studies relied on a distress scale instead of a stress scale, which might not report stress accurately, especially for first-year students [6,18]. These studies only included medical students of specific academic years, and none of them reported students’ perceptions of the factors contributing to their stress. The correlation between stress levels and students’ general health was not previously reported.

This study aims to identify the relationship between stress levels, students’ general health, and academic performance. We also aim to explore students’ perceptions of factors contributing to their stress. We suggest solutions and coping strategies to prevent stress in medical students.

## 2. Methods

### 2.1. Study Design

This is a non-experimental, cross-sectional study designed to find the possible factors contributing to stress among medical students and correlate these factors with the students’ academic performance and general health. The Institutional Review Board approved this study in March 2021.

### 2.2. Participants and Procedure

Medical students from different cities were randomly invited to participate in this online survey. Our participants consisted of 421 male and female medical students studying in one of the colleges of medicine in Saudi Arabia in different academic years (first year to internship year). The survey was distributed to medical students through social media platforms (Twitter (Twitter, Inc., San Francisco, CA, USA), WhatsApp (WhatsApp LLC, Menlo Park, CA, USA), and LinkedIn(LinkedIn Corporation, Sunnyvale, CA, USA).

The survey included an introductory statement describing the study’s aim and ensuring that participation was voluntary with complete anonymity. It also stated that they could withdraw from the study at any time. A 23-item self-administered online survey using Google Forms^®^ (Google LLC, Mountain View, CA, USA) was prepared and administered via Google. The survey was available from September 2021 to December 2021. Six participants were invited to pilot-test the initial draft survey to validate it; minor modifications were made based on their feedback. Then, the survey was electronically distributed. A call for participation in this survey was sent three times to maximize the response rate.

### 2.3. Measures

The survey included both open-ended questions and closed-ended questions assessing the following:

#### 2.3.1. Academic and Participant Characteristics

Closed-ended questions were provided to collect participants’ personal and academic characteristics, including gender, age, students’ grade point average (GPA), and year of study.

#### 2.3.2. Medical Characteristics

Students provided their medical information, which included the following: blood type (A, B, AB, and O), the presence of disease (comorbidities), previous or current COVID-19 infection, and weight and height to find the body mass index (BMI) (below 18.5 is underweight, 18.5–24.9 is normal, 25–29.9 is overweight, and 30 and above is obese).

### 2.4. Stress Level

Stress levels were measured using the Perceived Stress Scale (PSS), which is a 10-point validated stress scale to estimate general stress levels and individuals’ self-perceptions of their stress (Cronbach’s alpha evaluated at >0.70) [20,21]. Each question was scored using a range from 0 to 4. The final scores of the PSS range from 0 to 40, where a total score of 1–13 indicates low stress, 14–26 indicates moderate stress, and 27–40 indicates severe stress. The PSS was implemented in the survey to measure students’ general stress levels.

Additional questions were deployed regarding pandemic-related and academic-related stress to determine their contribution to stress in medical students. Moreover, open-ended questions were provided to identify sources of stress experienced by medical students, which were later categorized into themes according to their responses.

### 2.5. Statistical Analysis

Closed-ended questions: The statistical tests were performed using the Statistical Package for Social Science (SPSS) software version 26 (IBM Corp. 2019, Armonk, NY, USA). Descriptive statistical analysis was used to analyze the items included in the survey, such as participants’ demographics and other descriptive outcomes. Responses were presented as frequencies and percentages.

Chi-square and Fisher’s exact tests were used to compare responses between variables in different categorical measures. One-way ANOVA was used to compare responses between students of different education levels (first year to internship) with their medical characteristics.

For data that were not normally distributed (Shapiro–Wilk normality test), Mann–Whitney analysis was used to compare two groups, for example, academic performance with COVID-19 infection. The *p* values that are equal to or less than 0.05 were considered significant.

Open-ended questions: In the present study, data saturation is a point at which no new themes were created. The hybrid structure of our survey, with closed and open-ended questions, provided flexibility to apply quantitative and qualitative analytic methods for analysis. After gathering the results, the quantitative data were tabulated, and the textual responses were collated for further qualitative analysis using a thematic analysis approach to identify themes.

## 3. Results

The survey was available for three months (September 2021 to December 2021). Within the thematic analysis, we examined the recurrence of specific stress-related themes in the responses, which were categorized as the following: (i) general stress, (ii) academic stress, and (iii) pandemic stress. After reaching 400 participants, there were no new themes generated. Therefore, it was deemed that the data collection had reached a saturation point. We continued data collection for 21 more participants to ensure and confirm that no new themes were emerging.

### 3.1. Demographics

Our study included 421 medical students of all medical years from different universities in Saudi Arabia (both public and private universities). About 70% were clinical-year medical students, including fourth-year, fifth-year, and internship students. A student’s GPA was measured by a 5-point system. Most students’ (76.8%) GPAs were equal to or above 4 out of 5 points. Table 1 shows the details of the study demographics.

### 3.2. Medical Characteristics

Table 2 shows the medical characteristics of our sample. Half of our sample’s body mass index (BMI) was normal, and about a quarter was overweight. Students were asked if they had any comorbidities, including heart disease, migraines, allergies, eating disorders, bowel disorders, asthma, hypertension, diabetes, or blood disorders. About 18% have one or more comorbidities.

### 3.3. Stress Level

According to the Perceived Stress Test (PSS), most medical students (93.6%) were experiencing moderate to severe stress. About 31% reported that their stress increased during the COVID-19 pandemic. Table 3 shows the stress profile of our sample.

### 3.4. Stress in Medical Students

**Figure 1:**
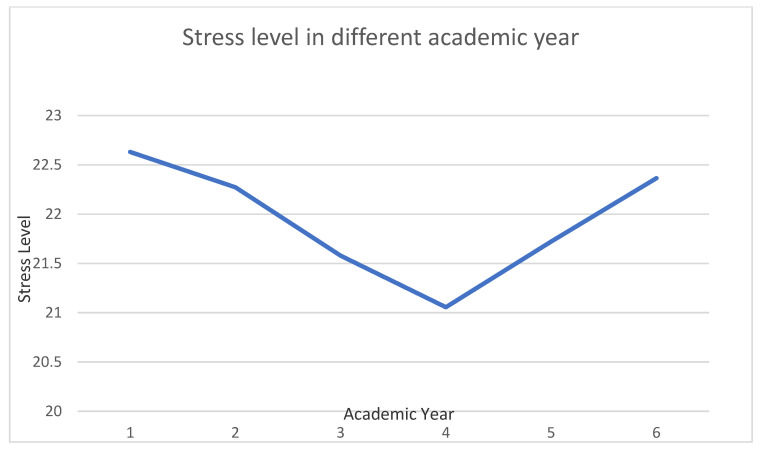
Shows the stress levels of medical students each year. First-year students are more stressed than other students (*p* < 0.05). Stress decreases each year except for the internship students (sixth-year medical students), where stress significantly increases (*p* < 0.05).

Most students were experiencing moderate to severe stress, which increased during the COVID-19 pandemic. Figure 1 shows first-year and internship students were significantly more stressed than second, third, fourth, and fifth-year students (*p* < 0.05). Moreover, students with higher GPAs were more stressed than those with lower GPAs (*p* < 0.001). Similar stress levels were experienced in males and females with different BMI levels. Linear regression analysis showed that stress was negatively correlated with students’ age (*p* < 0.05).

Medical students with comorbidities were significantly more stressed than healthy students, and their stress levels increased during the COVID-19 pandemic (*p* < 0.05). Pandemic-related stress was not significantly correlated with other variables such as GPA, academic year, general stress level, and age.

### 3.5. Medical Characteristics with Academic Performance

Most medical students were healthy with no comorbidities (81.7%). The most common comorbidity was asthma (6.2%). Medical students with comorbidities had significantly lower BMIs (mean 22.6 ± 4) than healthy students (mean 25 ± 6) (*p* < 0.05). Students with comorbidities had lower GPAs (lower by 4%) than healthy students. Other medical characteristics such as BMI, blood type, and COVID-19 infection did not significantly affect students’ academic performance.

### 3.6. Student Perceptions of Factors Contributing to Their Stress

An open-ended question was provided to identify the major stress factors that medical students experience according to their point of view.

Thematic analysis resulted in three main stress categories:

(i) General stress:

This category represented 25% of responses and included responses related to general fear and financial, social, personal, and family issues.

For example:

- Family and social obligations are overwhelming.
- No time for social activities, and a lack of ability to change the surrounding environment.
- Scared of the indefinite future.
(ii) Academic stress:

This category represents 34% of responses, including stress related to studying and academic performance, such as distance learning, grades, long studying time, lower GPA, less clinical exposure, exam stress, and frequent changes in decisions regarding teaching and examination procedures and timings.

For example:

- Forced to adapt to unpredictable governmental and university decisions regarding examination and teaching.
- Not knowing what to expect and continuous changes in school schedule and academic plans in general.
- Not enough clinical exposure in clinical years of medicine.
(iii) Pandemic-related stress:

Even though this category represents 41% of stress expressed by medical students in our study, most of it was related to academic stress.

For example:

- Not enough clinical exposure due to social distancing.
- What if I get infected during exams?
- The effect of the quarantine during the pandemic on my grades.
- What if I get the infection? Then my life must be withheld during the quarantine, which may impact my GPA.

## 4. Discussion

Stress among medical students has been recently recognized as a factor affecting students’ wellbeing and education. In this study, medical students’ stress levels (moderate 73.6% and severe 20%) were higher than the previously reported levels of 63% [6,18,22].

Except for the internship students, stress levels decrease as students progress academically. Similar results were previously reported, where stress was significantly reduced with each academic year. Still, these studies did not include internship students, had only female students, or used a distress scale instead of a stress scale, which might not reflect accurate stress levels, especially in first-year students [6,18]. Higher rates of stress experienced by first-year students are understandable since new curricula and teaching methods are introduced to them, which become more familiar as they progress in their education. Students adapt by developing coping skills each year, which decrease stress over time [18]. Social pressure from high expectations of students’ families and more social isolation with less time spent with family and friends increases their stress.

Positive coping strategies effectively reduce stress in medical students, but these strategies are ineffective in the final years of education [23]. In our study, the stress curve increases again between the last year and the internship year. This might be because of increased clinical obligations and expectations, which added physical, mental, and emotional pressure on internship students. In addition, internship students expressed fear of inadequate clinical training, and the competition for residency acceptance has increased due to limited training positions during the COVID-19 pandemic.

Physiological responses to stress affect students academically and physically, leading to health disorders, depression, and sleeping disorders at a young age, and might increase the risk of substance abuse [12,24]. Although comorbidities were reported in only 18% of our sample, we found that most (57%) were reported in fifth-year and internship students. Most reported disorders were migraines, hypertension, asthma, and irritable bowel syndrome, which might be triggered by stress. We also found that students with comorbidities are more stressed, but due to the nature of our study, we cannot predict a cause-and-effect relationship between stress and these disorders among our sample.

Students’ GPAs are affected by stress [18]. Older medical students with higher stress levels are most likely to have poor academic performance [22]. Interestingly, we found that students with higher GPAs are significantly more stressed than those with lower GPAs. This might be caused by stress related to the pandemic; students with higher GPAs in our study were more concerned with limited clinical exposure and the effect of the pandemic on their learning experience. This critical finding indicates that academic advising and psychological support should not be limited to students with low GPAs.

The COVID-19 pandemic has likely contributed to the high stress levels experienced by students in our study. During the pandemic, stress levels in the general population increased three-fold, which was also observed in our study, as 31% of students reported that their increased stress levels were pandemic-related [25]. Our findings are similar to a previous study that reported a high prevalence of stress (88%) among medical students during the COVID-19 pandemic [26]. Although most students reported being stressed due to the COVID-19 pandemic, they were mainly concerned about its effect on their education. For example, the stress experienced by the clinical-year students was related to lower clinical exposure, which might affect their future clinical skills and acceptance into residency programs. In contrast, preclinical students’ stress was focused on general stress, such as socializing with friends and family. A recent study examined the relationship between physiological and psychological health and academic stress during the COVID-19 pandemic and found that academic stress and fear of COVID-19 infection negatively impact students’ physiological and psychological well-being. However, a causal relationship was difficult to determine due to their study design, which was similar to ours. A longitudinal study is recommended to reflect the causation of these factors. [27]. Taking special care of medical students during pandemics using innovative means such as virtual counseling is crucial for their health and learning experience.

Stress that medical students experience should be carefully managed to achieve better education, clinical training, and patient care. However, traditional counseling is ineffective for medical students in coping with stress due to difficulty setting up appointments, stigma, and confidentiality issues. Therefore, a non-traditional alternative to counseling is recommended [8]. Embedding stress management and coping skills courses into the curriculum and creating a stress-coping initiative for medical students should be encouraged [23]. A counseling hotline with flexible timings is also suggested to provide coping strategies for early intervention.

## 5. Conclusions

The physiological effects of stress lead to serious health consequences. Students experience high stress levels in their final educational years, which might increase the risk of health issues and low academic performance. It is essential to innovate stress-coping strategies specially designed for medical students and mandatorily provided by all medical colleges and to educate students on the effects of stress on their health.

## 6. Limitations

This was a cross-sectional study based on self-reported data by medical students. Therefore, potential reporting bias is possible. Due to this study’s nature, it is impossible to determine a cause-and-effect relationship between stress and health status. Moreover, bias might be due to students’ mixed emotions between health and academic factors during the pandemic. It is essential to conduct a cohort study to examine students’ health status and academic performance after the pandemic.

## Figures and Tables

**Table 1:** Demographics.

Variable	*n* (%)
Gender	
Female	211(50.2)
Male	209 (49.8)
Age	
18–20 21–23 24–26	50 (11.8) 270 (64.2) 101 (24)
Year	
1	19 (4.5)
2	33 (7.9)
3	76 (18.1)
4	71 (16.9)
5	158 (37.6)
Internship	63 (15)
GPA	
5	14 (3.3)
4.5–4.99	215 (51.2)
4–4.49	97 (23)
3.5–3.99	59 (14)
3–3.49	17 (4)
2.50–2.99	11 (2.6)
2–2.49	6 (1.4)
Below 2	2 (0.5)

**Table 2:** Medical history.

Variable	*n* (%)
BMI	
Underweight	45 (10.7)
Normal	216 (51.2)
Overweight	104 (24.8)
Obese	56 (13.3)
Comorbidity	
Yes	77 (18.1)
No	344 (81.9)
Blood type	
O	181(43.1)
B	70 (16.7)
A	103 (24.5)
AB	24 (5.7)
COVID-19 infection	
Yes	86
No	334

**Table 3:** Stress profile.

Variable	*n* (%)
No stress	5 (1.2)
Mild stress	22 (5.2)
Moderate stress	309 (73.6)
Severe stress	85 (20)

## Data Availability

All research data are presented in this paper.
